# Radiofrequency ablation for primary hyperparathyroidism and benign thyroid nodules

**DOI:** 10.1002/kjm2.12919

**Published:** 2024-12-10

**Authors:** Yu‐Hsiang Lin, Tzu‐Yen Huang

**Affiliations:** ^1^ School of Medicine, College of Medicine Kaohsiung Medical University Kaohsiung Taiwan; ^2^ Department of Otorhinolaryngology—Head and Neck Surgery Kaohsiung Medical University Hospital, Kaohsiung Medical University Kaohsiung Taiwan; ^3^ Department of Otorhinolaryngology, School of Post‐Baccalaureate Medicine and School of Medicine, College of Medicine Kaohsiung Medical University Kaohsiung Taiwan; ^4^ Department of Otolaryngology—Head and Neck Surgery Kaohsiung Medical University Gangshan Hospital, Kaohsiung Medical University Kaohsiung Taiwan

Radiofrequency ablation (RFA) is a well‐established treatment for benign thyroid nodules, and it is a minimally invasive alternative to surgery. Recently, the application of RFA for primary hyperparathyroidism has emerged as a novel and promising treatment option.^1^ However, synchronous treatment of both benign thyroid nodules and primary hyperparathyroidism with RFA has rarely been reported, highlighting the significance of this case report in exploring the efficacy and feasibility of combined RFA therapy for these conditions.

A 63‐year‐old woman with a history of thyroid goiter was incidentally found to have hypercalcemia during a routine health examination, and further ^99m^Tc‐methoxyisobutylisonitrile (^99m^Tc‐MIBI) scan revealed a 1.8 cm primary parathyroid adenoma in the left inferior thyroid bed. The patient also complained a foreign body sensation while swallowing and was therefore referred to our clinic. Thyroid ultrasound showed a mildly hypoechoic parathyroid adenoma without vascularity, measuring 3.2 × 1.2 × 0.9 cm, and multiple thyroid nodules with benign cytology in the left lobe (Figure 1A). The patient experienced compression symptoms when the ultrasound probe was pressed on the thyroid nodule. Her baseline parathyroid hormone (PTH) level was 174.5 pg/mL. The patient opted for RFA treatment.

**FIGURE 1:**
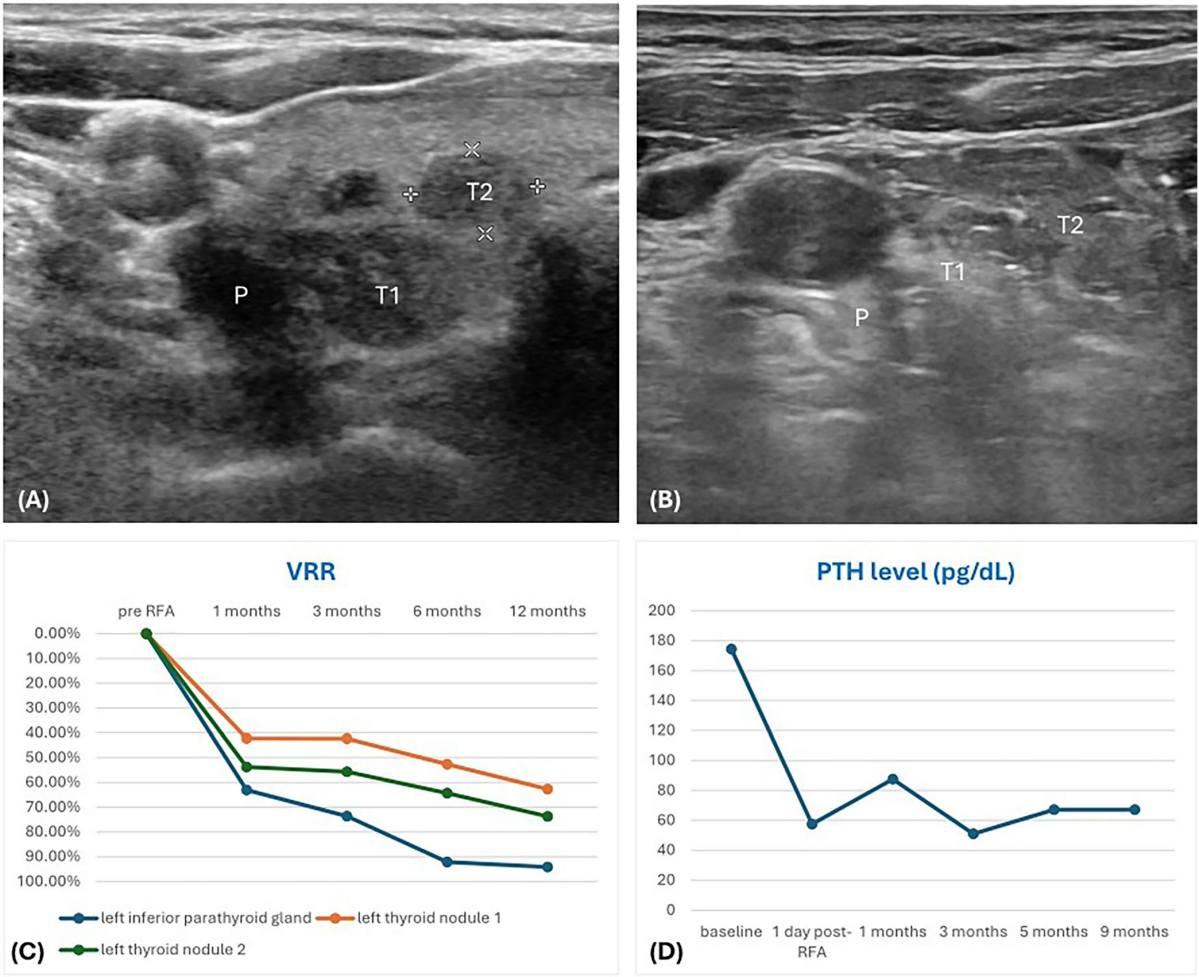
(A) Pre‐RFA ultrasound examination. Thyroid nodules (T1 and T2) and parathyroid adenoma (P) were marked. (B) Post‐RFA ultrasound showed hyperechoic feature. (C) VRR during the follow‐up period. The post‐RFA VRR of parathyroid adenoma at 12 months was 94.1%, and that of T1/T2 thyroid nodules was 62.7%/73.7%, respectively. (D) PTH level. The baseline level was 174.5 pg/mL. The post‐RFA PTH level at 9 months was 67.2 pg/mL. PTH, parathyroid hormone; RFA, radiofrequency ablation; VRR, volume reduction ratio.

Since the symptomatic thyroid nodules were adjacent to the thyroid capsule and the parathyroid gland was closely attached, it was evident that the thyroid nodules would be in the path of the parathyroid gland during the RFA procedure. To ensure safety, hydrodissection between critical structures except for the attached region was carefully performed, allowing the parathyroid adenoma and thyroid nodule to be treated as a single ablation zone through synchronous ablation. A 5‐mm RFA active tip was used with the moving shot technique, set at a power range of 20–30W, delivering a total energy of 9702 WS in 12 min. No complications were noted during the procedure. There was a significant reduction in the volume of the parathyroid gland and thyroid nodules during the follow‐up period (Figure 1B,C), as well as the PTH level (Figure 1D).

RFA is an alternative, nonsurgical, and minimally invasive treatment for thyroid and parathyroid disease. Indications include symptomatic thyroid nodules with 1–2 benign cytology results, without the need for histopathology report.^2^

The selection of the needle's active tip is crucial for parathyroid RFA, as once the energy spreads beyond the parathyroid gland, it can cause severe injuries involving the vagus nerve, muscles, or vasculature, which is more severe than in thyroid RFA. Generally, smaller active tips are preferred for parathyroid ablation because of the less unpredictable energy spread.^3^

While penetrating the thyroid capsule is necessary for effective treatment of the parathyroid gland, this procedure increases the risk of thyroid rupture and damage to surrounding structures, since voice change, hemorrhage, and nodule rupture are reported as the most common complication during thyroid RFA.^3^, ^4^, ^5^ To mitigate this risk, it is essential to consider the application of hydrodissection to prevent thermal spread and protect adjacent tissues, which is also necessary for thyroid ablation.^3^, ^4^ In our case, the operator needed to perform hydrodissection of the carotid sheath and strap muscles. However, there is no need to intentionally separate the thyroid capsule and parathyroid gland, since increasing the moving shot distance can reduce energy dissipation, minimize repeated punctures, and allow for more effective energy application to target lesions.^2^ In this case study, no complications were observed following the RFA treatment, and the patient experienced significant symptomatic relief during follow‐up.

In conclusion, synchronous RFA using a smaller active tip for thyroid nodules and parathyroid adenoma without separating the thyroid capsule is a potential treatment with several advantages, including minimal invasiveness, reduced tissue damage, and effective energy application, making it a promising option.

## FUNDING INFORMATION

The study was supported by the grants from National Science and Technology Council, Taiwan (NSTC 113‐2314‐B‐037‐035), and Kaohsiung Medical University Hospital, Kaohsiung Medical University (KMUH112‐2R51).

## CONFLICT OF INTEREST STATEMENT

The authors declare no conflict of interest.

## ETHICS STATEMENT

Approval for retrospective data collection/secondary use of health information was obtained from the Institutional Review Board of Kaohsiung Medical University Hospital (KMUHIRB‐E(II)‐20240332).
